# Meckel Diverticulum Causing Intestinal Volvulus

**DOI:** 10.1155/2020/8872668

**Published:** 2020-09-25

**Authors:** Liviu Musteata, Raouf Fayisall Geraldo, Hugues Ndasu Matendo, Aurélie Reitz, Valériu Krasovski, Valentin Nitu, Thierry Gastaud, Fabrice Cattan

**Affiliations:** ^1^Visceral Surgery Department, Hospital Center of Moulins-Yzeure, France; ^2^General Surgery Department, Faculty of Health Sciences, University of Lome, Togo

## Abstract

Intestinal volvulus is rare and responsible for upper bowel obstruction. They occur more frequently in a patient with abdominal surgery history. We report a case of small intestine volvulus on Meckel diverticulum, which occurred in a 21-year-old patient, with no history of laparotomy. The diagnosis confirmation was intraoperative, and the management consisted in a segmental resection of the small intestine with immediate anastomosis. The postoperative follow-up was good. This case underlines the scarcity and the severity of this presentation which therefore requires appropriate care in order to improve the prognosis.

## 1. Introduction

Small intestine volvulus is defined by the twist of the bowel around their vascular axis. It results in an upper obstruction presentation due to strangulation. It requires a timely management in order to have a positive impact on the patient's health. Usually, the onset of volvulus is due to the presence of abdominal adhesions in patient with abdominal surgery history. We report a case of small intestine volvulus seen in a patient without any surgical history. The objective is to describe the circumstances of diagnosis and the management and to do a review of the literature.

## 2. Patient and Observation

**Figure 1:**
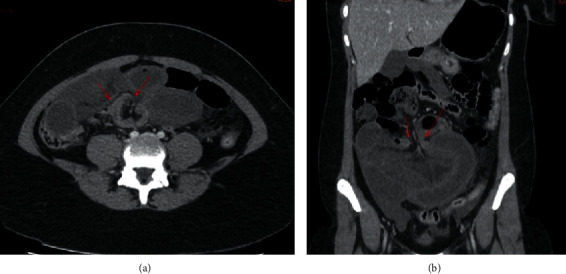
Abdominal CT scan injected in the late phase, showing a fluid dilation of the small intestine and a short junction syndrome in the periumbilical area with no image of “small bowel feces” upstream, reflecting a volvulus, with thick and poorly enhanced digestive walls: (a) axial section; (b) frontal reconstruction.

A 21-year-old female was admitted in the emergency department for crampy abdominal pain characterized by an acute onset, localized in the umbilical quadrant of the abdomen, evolving for two hours. It was associated with bilious vomiting, abdominal distension, and absence of gas. The patient had no abdominal or pelvic surgery history. At physical examination, she had fever (38.5°C) and tachycardia (108 ppm). The abdomen was distended and painful with tenderness localized on the umbilical area. The biological analysis revealed leukocytosis at 17 600/mm^3^ and increased lactate level at 4.7 mmol/L. The serum *β*-HCG level was normal, so was the CRP, ionogram, and the lipase rate. The liver and kidney functions were normal. A pain medication was started made of intravenous analgesic (paracetamol 1 g, nefopam 20 mg, and morphine 3 mg subcutaneously), but the pain was persistent. No plain radiology was performed. A contrast-enhanced CT scan of the abdomen and pelvis revealed a closed-loop small bowel obstruction (Figure 1).

**Figure 2:**
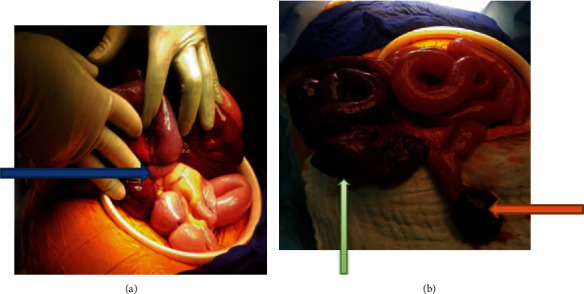
Intraoperative image showing Meckel's diverticulum attached to the mesentery (a, blue arrow), necrotic at its tip (orange arrow), and necrotic intestinal loops (b, green arrow).

A laparotomy was performed in emergency after premedication (nasogastric tube, bladder catheter, and intravenous fluid). At the opening of the peritoneum, a blood-tinged fluid and a very tight volvulus of the small intestine with intestinal necrosis extending about 25 cm at 60 cm from the ileocecal junction were found. Meckel diverticulum was found at the basis of the volvulus, fixed on the adjacent mesentery (Figure 2(a)) measuring 15 cm. It was localized at 80 cm of the ileocecal junction and necrotic at its distal end (Figure 2(b)). There was no omphalomesenteric duct identified.

The procedure consisted in a segmental resection of the area of necrosis and Meckel's diverticulum followed by a side-to-side anastomosis and peritoneal lavage. The postoperative follow-up was simple, and the patient was discharged to home on postoperative day 10. Histolopathological examination of the resected specimen revealed ileal ischemic necrosis on Meckel diverticulum without gastric heterotopia and without signs of malignancy.

## 3. Discussion

The small bowel volvulus requires emergency treatment because of the risk of generalized peritonitis secondary to bowel perforation. It occurs suddenly with rapid evolution to intestinal necrosis. Biological signs of an inflammation are often found. Management must be appropriate to improve the vital prognosis. The occurrence of the volvulus implies the presence of a fixed point for the rotation. In this way, adhesions represent the most common cause, especially in patients with previous abdominal surgery. This is not the case for the patient in our study. We report a case of small volvulus on Meckel's diverticulum.

Meckel's diverticulum is the embryonic remnant of the yolk duct. It is rarely found in adults but is more frequent in the male subject [1]. When present, it sits in 50% of the cases between 10 cm and 100 cm from the Bauhin valve; its average dimensions are 2 cm in diameter and 5 cm in length [2]. Often asymptomatic, it is discovered as a complication: digestive hemorrhage, diverticular perforation, cancer, diverticular volvulus [3], and Meckel's diverticulitis [1, 4]. Small bowel volvulus complicates 5.5% of Meckel's diverticulum [5]. The main mechanism described is the persistence of a true omphalomesenteric canal which constitutes an anchor point for the rotation of the handles [1]. In the case reported herein, this duct was not found. Meckel's diverticulum was found fixed on the mesentery. What could constitute the anchor point of the rotation of the slender handles, at the origin of the volvulus? This mechanism has not yet been described. In addition to the presence of omphalomesenteric canal, etiological factors have been reported. Fontenot et al. described a case of small bowel volvulus on Meckel's diverticulum adhering to the laparoscopic trocar insertion port [6]. In addition, there were nine cases of internal hernia on Meckel's diverticulum, performing mechanical occlusion [7].

However, a few cases have been reported in the literature with no objectified fixation point. In 2015, a 32-year-old patient with no previous surgical history was admitted for an intestinal obstruction. The abdominal CT scan objectified images in favor of a small volvulus. Laparoscopy for diagnostic purposes made it possible to find volvulated but viable small handles on Meckel's diverticulum (neither volvulated nor necrotic). There was no objectified omphalomesenteric duct [8]. In 2019, a second case was reported in a 30-year-old patient, admitted for an upper intestinal obstruction. The CT scan was not performed in an emergency, and management consisted of emergency laparotomy. A 75 cm intestinal necrosis was discovered on an 8 cm Meckel diverticulum, 60 cm from the ileocecal junction [9]. Management is surgical. A gesture of intestinal resection is always performed, taking away the diverticulum. Intestinal continuity is generally restored immediately, and the follow-up is often simple [8, 9]. The preferred approach is laparoscopy, which has a dual diagnostic and therapeutic value [10]. The choice of laparotomy in our case was due to the surgeon habits.

The prognosis is generally good, and the follow-up surveillance is based on clinical sign assessment (transit, temperature, and clinical examination of the abdomen).

## 4. Conclusion

Meckel's diverticulum is rare and often asymptomatic. Its discovery results from the occurrence of complications including diverticulitis and intestinal obstruction. The diagnosis is intraoperative with an associated intestinal resection gesture. This case highlights the rarity of small hail volvulus on Meckel's diverticulitis.
